# Surrogate endpoints in cost-effectiveness analysis for use in health technology assessment – a qualitative analysis

**DOI:** 10.1017/S0266462326103791

**Published:** 2026-08-07

**Authors:** Laura Flight, Fatima Salih, Shane D Collins, Phyo Aung, Michael Coory, Daniel A Ollendorf, Marina Richardson, Zoe Garrett

**Affiliations:** 1 National Institute for Health and Care Excellence, UK; 2 Department of Epidemiology and Preventive Medicine, Monash University, Australia; 3 Mater Research Institute, Faculty of Medicine, University of Queensland, Australia; 4 Institute for Clinical and Economic Review, USA

**Keywords:** surrogate endpoint, surrogate outcome, health technology assessment, health economic model

## Abstract

**Objectives:**

The use of surrogate endpoints poses challenges to health technology assessment (HTA) agencies and payers when informing the clinical and cost-effectiveness of health technologies. Decision makers often need to make recommendations in the absence of well-established evidence on their validation, particularly when the surrogate endpoint is novel without clear links to the longer-term outcome. This study explored the guidance needs of HTA agencies and identified how they have considered and dealt with surrogate endpoints in cost-effectiveness analysis.

**Methods:**

A qualitative study was used to explore the professional experiences of international HTA staff and their collaborators. Data were collected using three focus groups with 29 participants representing 20 HTA agencies or associated organizations. Framework analysis was used to identify themes from the transcripts.

**Results:**

HTA agencies frequently see submissions featuring surrogate endpoints. Issues they face include definitional concerns, absent or weak evidentiary links, and resource-intensive validation methods. Versatile guidance that is mindful of these challenges, such as those faced with rare diseases, is required. This would help to clarify expectations and help ascertain the required information from industry for economic modeling, on how to present the uncertainty arising from surrogate endpoints, and how to appropriately report the findings.

**Conclusions:**

Decision making using surrogate endpoints is a frequent and challenging problem faced by HTA agencies. These results provide the first-hand experiences and reflect the needs of HTA agencies globally. The resulting recommendations and considerations for the use of surrogate endpoints in economic modeling should benefit HTA agencies moving forward.

## Introduction

Health technology assessment (HTA) is a “multidisciplinary process that uses explicit methods to determine the value of a health technology at different points in its lifecycle. The purpose is to inform decision-making in order to promote an equitable, efficient, and high-quality health system” (1). Often, HTA takes a long-term perspective that assesses, using evidence of clinical and cost-effectiveness, the future impact in terms of health outcomes and costs of introducing a new technology in the healthcare system (2).

Surrogate endpoints are biomarkers or other intermediate outcomes that are correlated with or are expected to reliably predict a clinical benefit or harm (3). This type of endpoint is of relevance to many HTA agencies because of their increasing use in regulation (4–6) which can result in a lack of data on long-term effectiveness when a new health technology enters the market (7).

Some HTA agencies have published methodological guidance on the acceptability of using surrogate endpoints to inform decision making (4,8). However, the level of detail varies widely, and there is limited guidance on validation and considerations for economic modeling (4;8;9). Furthermore, despite the extensive published general methodological guidance on conceptualization of economic models and evidence synthesis, there remains a lack of clarity on how to address surrogate endpoints specifically when conceptualizing an economic model to inform HTA (4;7;10;11).

In this context, a group of HTA agencies collaborated to produce recommendations about using surrogate endpoints in health economic models to inform HTA decision making. The project activity was led and coordinated by the National Institute for Health and Care Excellence (NICE) who formed a working group that included the following organizations:

- Australian Government Department of Health and Aged Care
- Canada’s Drug Agency (CDA-AMC)
- the Institute for Clinical and Economic Review (ICER), USA
- the National Health Care Institute (ZIN), Netherlands
- Colombian Institute for Technology Assessment in Health (IETS)
- Rubix Health, USA

Working group members led on different tasks, and the results from each task were then used to determine best practices in model conceptualization for cost-effectiveness analysis involving surrogate endpoints. The final set of recommendations is designed to be used alongside existing economic modeling guidance and includes considerations around definition, justification, adoption, statistical validation, incorporation, reporting, and approaches to quantify and present uncertainty, when using a surrogate endpoint (4).

This paper describes one of the activities that informed the final recommendations. The objective of this study was to identify how HTA agencies have considered and dealt with the uncertainties around surrogate endpoints in their technology evaluations, with a focus on economic evaluation.

## Methods

Data were collected for this study using focus groups, as this allowed for interaction between participants and open discussions (12). Three focus groups were conducted globally. The focus group for participants in the Americas was led by DO and MR, the European focus group was led by ZG, and MC and PA led the focus group in the Asia-Pacific region. NICE staff (LF, FS, SC) attended and supported each group.

HTA agencies were identified that were known to use, develop, or perform health economic evaluations as part of HTA. This included established HTA agencies and newly established HTA agencies with global geographic representation. The agencies then identified members of staff who had a technical or operational role and were involved in the evaluation of technologies. They also needed to be able to provide information on their experience of their agency on the use of surrogate endpoints for decision making.

Participants were invited through existing contacts of the working group. Contacts were approached via email and asked to forward to relevant staff. All participants were sent an information sheet and asked to complete a consent form before joining the focus groups.

A document outlining the aims of the focus group and four case studies involving the use of surrogate endpoints was sent to each participant prior to the meeting to help facilitate the discussions. This included anticipated questions to allow participants time to consider their response and research previous actions within their agency. The case studies included metabolic-associated steatohepatitis (MASH), obesity, Duchenne muscular dystrophy (DMD), and multiple myeloma. These topics were selected by the working group to reflect a range of surrogate endpoints and topics that participants may have experience of or an interest in.

- Obesity was chosen as a topic that has well-established surrogate endpoints with validated correlation to patient-centric outcomes that are likely to have been seen by all participants.
- Minimal residual disease (MRD) in multiple myeloma was selected as a less well-established surrogate endpoint that fewer participants will have experience of but is likely to be seen in HTA in coming years.
- DMD was chosen as a rare disease that utilizes surrogate endpoints due to its small patient populations making lengthy clinical studies challenging.
- MASH was selected as a topic that is likely to be seen in coming years where surrogate endpoints are required to extrapolate over long periods of time.

The focus groups were divided into three parts: (1) open discussion asking participants to reflect on how their HTA agency handles surrogate endpoints and the issues they have encountered, (2) discussion of specific case studies, and (3) recommendations or advice they would give to health technology developers. Discussions were conducted online via Microsoft Teams, video recorded, and transcribed using Microsoft Teams and then checked and edited by a member of the team. Focus groups lasted approximately 2 hours.

Ethics approval was obtained from Newcastle University (reference number: NICE 42449/2023).

### Outline of analysis

Transcriptions were imported into Lumivero (2023) NVivo (Version 14) www.lumivero.com (13). A framework analysis approach was adopted for this study (14). After familiarization with the data, an initial thematic framework was developed based on the research aims and discussions with the working group. This consisted of four key points in the data (themes):

1. Guidance on the use of surrogate endpoints in economic modeling for HTA
2. Issues when using surrogate endpoints in economic modeling for HTA
3. Consequences of using surrogate endpoints in economic modeling in HTA decision making
4. Suggested recommendations for using surrogate endpoints in economic modeling for HTA

Two people (LF and FS) then read one of the focus group transcripts independently, line by line assigning a code from the initial framework and noting any additional themes with no obvious place in the framework. The framework was then updated. LF applied it to the two remaining focus groups. All the data, from each focus group, relating to each code in the framework were collated into a framework matrix.

The results were interpreted in the context of the research aims and written into a draft report. The draft report was circulated to focus group participants and two further HTA staff who had consented to be part of the focus groups but were unable to attend on the day. They were given the opportunity to provide feedback and where appropriate this was incorporated into the analysis.

Once the feedback process was complete, a final framework was developed and used to structure the findings reported below. The four final themes were

1. Experience of surrogate endpoints in economic modeling for HTA
2. Challenges of using surrogate endpoints in economic modeling for HTA
3. Guidance on the use of surrogate endpoints in economic modeling in HTA decision making
4. Recommendations for future use of surrogate endpoints in economic modeling for HTA

## Results

A total of 29 participants took part in the study between May and June 2024, as summarised in Table 1. There were 20 HTA agencies or associated organizations representing countries from Europe, the Americas, and Asia-Pacific regions.

**Table 1.** Summary of the number of participants and agencies and associated organizations represented in the three focus groups Table 1. long description.

Focus group	Number of participants	Number of agencies/organizations
Europe	9	7
Americas	10	6
Asia-Pacific	10	7

### Experience of using surrogate endpoints in economic modeling for HTA

#### Defining surrogate endpoints

Participants suggested that there was a lack of clarity about the definition of a surrogate endpoint. Some participants viewed biomarkers and intermediate outcomes differently and, in some cases, did not consider them always to be a surrogate endpoint. For example, progression-free survival (PFS) is an intermediate outcome with a well-accepted link to survival in certain cancers; some participants did not initially talk about PFS as a surrogate endpoint.

#### Frequency of encountering surrogate endpoints in evaluations

Across the discussions, regardless of their definition of a surrogate endpoint, participants agreed that their use in HTA submissions was increasing, with upward of 50 percent of assessments using a surrogate endpoint. This was thought to be a consequence of expedited regulatory approvals and trials targeting patients with earlier stage disease and commonly seen in more innovative technologies or rare diseases, such as DMD.

*It makes sense that we’re seeing more submissions that are using either what we call a true surrogate or the intermediate surrogates. Because in oncology anyway, you’ve got all the treatments moving earlier and earlier. So adjuvant neoadjuvant space, you’re not going to have overall survival benefit demonstrated in the trial.*

European focus group participant

#### Handling the increase in use of surrogate endpoints to inform decision making

Participants recognized that many HTA agencies have limited guidance on how to handle surrogate endpoints in their methods manuals. In response to the increase in use of surrogate endpoints, some participants described additional internal documents that are used to inform their evaluations. Where internal guidance is available, it was said to be centered around providing justifications for the inclusion of the surrogate endpoint in the economic model and exploring different scenarios to better understand the uncertainty due to the surrogate endpoint. There was consensus among participants that it would be useful to have more detailed guidance for the evaluation of clinical effectiveness based on surrogate endpoints and for its translation to the assessment of cost-effectiveness.

#### Awareness of surrogate endpoints in the pipeline through early engagement with industry

Participants from HTA agencies that have early scientific advice services for technology developers highlighted the value of knowing about potential new surrogate endpoints in the pipeline through the advice requests. This gave them experience and knowledge of a surrogate endpoint before seeing it in a submission. For example, seeing how surrogate endpoints such as MRD are being used in the evaluation of multiple myeloma treatments.

*We do have a scientific advice programme where we support a sponsor in their very, very early stages of planning a trial. And it’s sometimes in that space we see what might happen in the pipeline five or six years down the line and in that space, we have seen sponsors who are at least planning for the primary or sometimes a composite a co-primary clinical endpoint to be […] NASH CRN scoring system.*

Americas focus group participant

### Challenges of using surrogate endpoints in economic modeling for HTA

#### Challenges when evaluating economic models that use surrogate endpoints

Participants reported that in their experience, economic models are designed around the surrogate endpoint, rather than the disease pathway, which makes model outputs difficult to validate and apply to other contexts. It was also highlighted that in some instances, the surrogate endpoint is embedded within the model structure in ways that can add complexity to their evaluation and make it challenging to separate the impact of the assumptions made about the relationship between the surrogate endpoint and the final outcome. An example of this is when a surrogate endpoint informs multiple transition probabilities that affect different outcomes, like the model used in ICER’s evaluation of resmetirom and obeticholic acid for NASH/MASH (15).

*Like the NASH model, […] It’s […] an indirect route through multiple transition probabilities that follow that surrogate relationship.*

Americas focus group participant

Participants highlighted that in HTA, intermediate endpoints that include a measure of function or symptoms and used as a substitute for a target outcome are considered as surrogate endpoints. This can include outcomes such as quality of life outcomes or adverse events. For example, in an evaluation of liraglutide for managing weight and obesity carried out by NICE in the UK, the final endpoint, cardiovascular benefit of liraglutide in the economic model was based on risk reduction based on surrogate endpoints hemoglobin A1c and blood pressure. Participants stressed that it is important that these outcomes are also validated if they are to be used in the economic modeling. As can occur in trials without surrogate endpoints, participants also identified that in some cases there was disconnect between how the surrogate endpoint was measured in the clinical trial and what was being modeled making it challenging to interpret and incorporate the evidence into the modeling.

### Challenges when assessing the uncertainty arising due to the use of surrogate endpoints

Due to the opportunity cost of decision making based on surrogate endpoints and the potential losses if an incorrect decision is made, uncertainty needs to be mitigated. Some of the HTA agencies use temporary reimbursement decisions or managed access when the evidence to support the use of a surrogate endpoint is lacking. For example, an HTA agency might give a temporary reimbursement or time limited recommendation along with the condition that further evidence is provided in three years’ time. This flexibility alleviates some of the issues that arise when evaluations are a “single decision at a single time point” *(*European focus group participant). Other participants cited using scenario analyses to explore the uncertainty in their economic models such as considering the best and worst values for the surrogate endpoint, removing the surrogate endpoint entirely from the model, and even using value of information analysis to identify and prioritize further research in areas where the further reduction in uncertainty associated with the use of the surrogate endpoint is worth the extra resources required to collect the data (16).

### Challenges when validating a surrogate endpoint

The study participants suggested that it was key to understand what evidence and previous decisions had been made in the same technology, for the same disease or using the same surrogate endpoint. This was especially true for those HTA agencies where a decision to recommend a technology cannot be reversed and so sets a precedent for future decision making. However, many participants felt that the requirements of some validation processes are complex, time consuming and costly for technology developers and the HTA agencies evaluating the evidence.

*The process involved in trying to validate a surrogate is complex and time consuming and it’s almost like a submission in itself just to do that.*

Asia-Pacific focus group participant

It was also felt that it is important to consider the validity of a surrogate endpoint in the context of how and why it is collected. Often HTA agencies receive evidence on the validity of a surrogate endpoint after it has been evaluated by a regulator. This means it is collected and presented to meet the regulator’s needs, and the HTA agency is unlikely to be able to contradict any conclusions drawn about a surrogate endpoint’s validity. The HTA agency will still need to see and understand this evidence for their own decision making, but this can be challenging in the face of a lack of transparent evidence of validity. For example, the FDA surrogate endpoint table does not provide the validated evidence used in their decision making process (17).

### Alignment in expectations between regulators, HTA agencies, and technology developers

There can be a lack of alignment in expectations between regulators, HTA agencies, and technology developers regarding the level of information required for decision making, especially relating to the surrogate endpoint. The evidence presented to regulators may not be sufficient to accurately represent a technology’s use in clinical practice if it is recommended by an HTA agency. For example, an HTA agency is often required to extrapolate the benefits and costs of a technology over a lifetime for use in an economic model.

The differing expectations are exacerbated by the lack of guidance on how to transparently report the evidence relating to surrogate endpoints. This makes it difficult to conduct thorough evaluations for a health technology and make robust recommendations. It was also identified as a stumbling block for many of the participants as it made it difficult to properly understand the evidence around the relationship between the surrogate endpoint and the final outcome.

*It’s almost like getting information like with little teaspoons.*

Americas focus group participant

### Guidance on the use of surrogate endpoints in economic modeling in HTA decision making

#### Approach to providing additional guidance to technology developers around use of surrogate endpoints to inform decision making

Participants highlighted the following aspects that should be considered when developing additional guidance around surrogate endpoints:

- Variation in capacity and resource of different HTA agencies: if technology developers are requested to provide extensive volumes of additional information, less-resourced HTA agencies may struggle to assess this information within strict timeframes.
- Flexibility of guidance to be applicable in different situations where surrogate endpoints may arise: if guidance is too stringent, it may not accommodate situations where specific evidence is lacking, so it is important to present a tiered approach based on aspects like availability of evidence. For example, in NICE methods guidance the EQ-5D is set out as the preferred measure of quality of life in adults. However, multiple options are provided that would be acceptable where this evidence is not available from a clinical trial, for example, using data from the literature or mapping from other quality of life measures (18).
- Consistency within an organization: there is a need to have an approach that can be consistently applied to all submissions within an HTA agency.
*There’s a consistency aspect if you have that flexibility to do something different every time, then what you get is an inconsistent approach every single time so that is not helpful to us and it’s really not helpful to those who submit their evidence to us.*

Americas focus group participant

- Consistency between organizations: greater consistency between HTA agencies would support them to be able to use each other’s technical work and analysis. However, participants receiving HTA submissions on a delayed timeline relative to other jurisdictions did identify instances where mature data were available, but technology developers had submitted earlier data based on earlier submissions to HTA agencies in other jurisdictions. Guidance needs to consider launch patterns and changes to data availability over time.

#### Importance of the context and relevance of a surrogate endpoint to patients

The participants suggested that the use of surrogate endpoints in rare and ultra-rare disease, such as DMD, can be challenging, and additional guidance would be helpful. The use of surrogate endpoints in this setting is increasingly common due to the novelty of treatments in this area; however, evidence to support a surrogate endpoint can be minimal due to the small population or the immature data collection in this setting and due to the absence of other treatments.

*The intermediate outcomes, we haven’t really assessed those outcomes before and until they’ve been assessed and […] had that clinical involvement, we don’t know whether or not they’re […] a surrogate for what we should be looking for.*

European focus group participant

In this situation, participants suggested that it is important to keep the focus on what matters to patients and to consider the broader context to the decision making. For example, is there an unmet need in this population, is this a rare disease with few treatment options, is this a life-threatening condition, is it a refractory population and resistant to previous treatments? One participant noted that their agency has developed a multi-criteria decision analysis (MCDA) framework that help them to consider criteria including effectiveness and severity of disease, especially in relation to rare diseases. Participants also mentioned discussions with patients and clinical experts. Another participant suggested they consider relevant regulator decisions that involved the surrogate endpoint.

#### Guidance on how to validate a surrogate endpoint for use in an economic model

Participants were keen to have more guidance on how surrogate endpoints should be validated, including appropriate methods and how this should be presented. They wanted to see a set of standards that outlined how the relationship between the surrogate and final outcome should be calculated at an individual and a trial level.

The use of real-world evidence (RWE) and later data cuts from clinical trials were suggested by participants as a potential way to collect more information to validate a surrogate endpoint. For example, a temporary or conditional recommendation could be made on the basis that further evidence is collected to support the surrogate relationship.

*If we have to be making a decision early that there is the opportunity in the future to be re looking at those, but based on real world evidence, based on observational data. And we should be putting in the infrastructure to look at that.*

European focus group participant

Although available evidence may be helpful in certain situations, there were concerns that in many situations data may be collected in a way that is not useful for developing the model and for the assessments of clinical effectiveness, especially if different in nature (heterogeneous populations and comparators) to the clinical trial evidence that may also be employed to develop the model.

For some HTA agencies, a literature review of clinical studies, quality of life studies, previous HTA reports, and economic models in the disease area is required to inform the evidence synthesis and economic model, but this was not the case for all. The suggestion was that guidance could outline how a literature review of evidence should be carried out and presented as part of the submission to support the use of a surrogate endpoint. This should include the clinical evidence underlying and justifying the use of a surrogate endpoint. On the limited occasions when there is a systematic review of evidence of a surrogate endpoint, participants said they still rely on discussions with clinical and public experts to interpret this evidence and provide their views on the surrogate endpoint.

Some participants were keen to stress that past economic modeling approaches and HTA decisions should not dictate future decisions but should help to inform them and so the evidence presented should be interpreted with this in mind and decisions reflect more up-to-date knowledge.

*Really you should build on what’s been done before. Not, carry on doing it.*

European focus group participant

#### Guidance to facilitate transparent reporting of evidence (setting expectations with technology developers)

The participants wanted to see recommendations that would facilitate better reporting of evidence from technology developers that would help them to understand the surrogate relationships, how the economic models had been built and help them to explore the uncertainties themselves. There was also a suggestion that transparent reporting is required from HTA agencies and modelers on how they are using the evidence from a surrogate endpoint, how it has been validated, and how this might affect the results. This could also include the procedure of modeling, the assumptions, the sensitivity analyses performed, their limitations, and their strengths. It was felt that guidance would help to align the expectations of both HTA agencies and technology developers during the submission and assessment process.

*I would want to see a transparent model that would allow me to make changes to the model and to test various scenarios.*

Asia-Pacific focus group participant

#### Database of surrogates

Multiple participants suggested that a “living library” of surrogate endpoints and their available evidence that is shared across HTA agencies could be useful alongside a guidance document. However, it was noted by other participants that this could be challenging due to the different contexts that surrogate endpoints can be applied even for the same active ingredient or indication.

*I know it’s very ideal, a living library of […] model relationships between surrogates and final outcomes would be lovely.*

Asia-Pacific focus group participant

### Suggested recommendations for future use of surrogate endpoints in economic modeling for HTA

Participants discussed recommendations that would support their work using surrogate endpoints in economic modeling for HTA. The suggested recommendations from the focus group participants are summarized in Table 2. These suggested points were considered in the development of the final recommendations, alongside the outputs from the wider project activities (4).

**Table 2.** Focus group recommendations for future use of surrogate endpoints in economic modeling for HTA used to inform the findings of the wider project (4) Table 2. long description.

Themes	Details
Recommendations for developing guidance on surrogate endpoints	Guidance should: be flexible and versatile so it can be applied to a wide range of scenarios, e.g., using a tiered approach or an algorithm that accounts for variability in factors such as availability of evidence reflect the varying structures and resources of HTA agencies globally. consider the different launch patterns of technology developers that mean some HTA agencies receive submissions later than others and hence receive different data due to changes in data availability over time. be mindful of areas where the evidence about the surrogacy relationship may be particularly challenging to inform, e.g., for new technologies for treating rare diseases.
Recommendations for developing an economic model using a surrogate endpoint	Recommendations in the development of an economic model that uses a surrogate endpoint should include Model conceptualization, design, and structure The model conceptualization should be based around the disease area rather than the surrogate. The economic model should take precedent into account but remain flexible, avoiding reliance on previous approaches and decision making if they are no longer appropriate or applicable to the current situation. The use of a surrogate endpoint in an economic model should be justified and supported by data including how the context of the clinical assessment supports the validity of the surrogacy relationship. The model should be structured to accommodate updates with future data cuts, allowing for the integration of mature evidence on surrogate endpoints, or replacing them with final outcomes as they become available. The model should ideally be built so that the effect of the surrogate endpoint can be removed to allow a full exploration of its impact on the results. For example, the effect of treatment on final outcomes via the surrogate endpoint and the duration of that effect should be parameters which can be explored within the model. Validation of the surrogate endpoint should include: Clinical expertise to understand the clinical relevance of the surrogate endpoint. A comprehensive review of the evidence on the relationship between the final outcome and the surrogate endpoint. Real-world evidence collection could be used as an option to validate the surrogacy relationship. Assessing uncertainty in the economic model. Test assumptions around relationship of surrogate endpoint to final outcome in scenario analyses, e.g., best and worst-case scenarios. Consider the use of advanced techniques such as value of information analysis to explore the benefit of reducing key uncertainties associated with the use of a surrogate endpoint.
Recommendations for reporting an economic model using a surrogate endpoint	Transparent reporting of an economic model using a surrogate endpoint supports understanding the evidence and uncertainties associated with the use of a surrogate endpoint in economic modeling.
Recommendations for further work	A database of surrogate endpoints and the evidence to support its use would help HTA agencies to validate their surrogate endpoints and potentially reduce the workload associated with the validation step. Such a database could build on the Food and Drug Administration’s Table of Surrogate Endpoints That Were the Basis of Drug Approval or Licensure (17).

## Discussion

Surrogate endpoints are increasingly used for regulatory purposes and with this there has been an increase in their use in HTA submissions (3). Surrogate endpoints have been identified as a challenge in the HTA of complex health technologies where data can be absent or unavailable for clinical outcomes (19;20). Additionally, for cell and gene therapies, 70 percent of HTA reports included the use of a surrogate endpoint (21), but validation is challenging (22–25). This study used focus groups with participants from HTA agencies and their related organizations to explore how HTA agencies consider surrogate endpoints in their evaluations and their needs for further guidance. Recommendations are summarized that aim to promote consistency within and between HTA agencies and clarify expectations between technology developers and HTA agencies.

The focus groups confirmed anticipated challenges and experiences of HTA agencies and their associated organizations such as the increased use of surrogate endpoints and the lack of clear guidance on validation. The focus groups also highlighted the tensions between the complexity and evidence requirements of current methods for validating surrogate endpoints with the resources available in many HTA agencies. The recommendations for better reporting of health technology evaluations using surrogates align with recent work encouraging and supporting the better reporting of the use of surrogates in trial protocols (26) and trial reports (27). Recognizing that the use of surrogate endpoints in economic modeling is related with their use in clinical effectiveness, the wider project activity included an update of a review published in 2020 (8). This means that final project recommendations build on existing guidance from HTA agencies and with methodological guidance published by the coordination group for the EU HTA regulation (28).

The topic of the use of surrogate endpoints in HTA is not a new one. The Horizon Europe COMED (Pushing the Boundaries of Cost and Outcome Analysis of Medical Technologies) project completed quantitative and qualitative work on this topic (29). However, as far as we are aware, this is the first qualitative study focusing on the perspective of staff at HTA agencies and their associated organizations on the use of surrogate endpoints in economic evaluation in HTA and their suggestions for recommendations.

There are a number of limitations with this study. The study does not capture the perspectives of all HTA agencies, but it captures a breadth of HTA agencies of different maturity and resourcing levels in different areas of the world. Four specific case studies were used to center the discussions; the majority of the discussions were focused on general experiences and challenges relating to the use of surrogates in economic modeling in HTA. It is not felt that the choice of case studies influenced the results obtained, and the findings are generalizable to all health technology evaluations. The focus groups each had a different facilitator, which could have led to variation in content and delivery of the questions and discussion. However, NICE staff attended all the focus groups, and the use of different facilitators may also have enriched the breadth of opinion.

## Conclusions

Decision making using surrogate endpoints is a frequent and challenging problem faced by HTA agencies. These results provide the first-hand experiences and reflect the needs of HTA agencies globally. Participants suggested that future guidance and recommendations on the use of surrogates in economic modeling for HTA should be flexible, adaptable, and consistent. Guidance should outline how to report methods and results transparently, include how to validate surrogacy relationship including methods, presentation, and minimum standards and consider patient needs and acknowledge scenarios where standards of evidence may be more challenging (e.g., rare diseases). The findings from these focus groups have been used with other project outputs to develop a set of recommendations to be used alongside existing economic modeling guidance and include considerations around definition, justification, adoption, statistical validation, incorporation, reporting, and approaches to quantify and present uncertainty, when using a surrogate endpoint (4).

## Long descriptions

### Table 1. Long description

The table consists of three columns and four rows including the header.

Column headers from left to right are: Focus group, Number of participants, and Number of agencies forward slash organizations.

Row 1: Europe focus group has 9 participants and 7 agencies forward slash organizations.

Row 2: Americas focus group has 10 participants and 6 agencies forward slash organizations.

Row 3: Asia-Pacific focus group has 10 participants and 7 agencies forward slash organizations.

Navigate back to Table 1..

### Table 2. Long description

The table is organized into four main thematic rows.

1. Recommendations for developing guidance on surrogate endpoints: Details specify that guidance should be flexible and versatile, using tiered approaches or algorithms. It must reflect global H T A agency structures and resources, account for varying technology launch patterns and data availability, and address challenges in rare disease evidence.

2. Recommendations for developing an economic model using a surrogate endpoint: This section is divided into three sub-points:

• i. Model conceptualization, design, and structure: Models should be disease-area focused rather than surrogate-focused, remain flexible to avoid outdated precedents, justify surrogate use with clinical data, accommodate future data updates, and allow for the removal of surrogate effects to explore impact.

• ii. Validation of the surrogate endpoint: Requires clinical expertise, comprehensive evidence reviews of the relationship between final outcomes and surrogates, and the potential use of real-world evidence.

• iii. Assessing uncertainty: Recommends testing assumptions via scenario analyses and using advanced techniques like value of information analysis.

3. Recommendations for reporting an economic model using a surrogate endpoint: Emphasizes transparent reporting to support the understanding of evidence and uncertainties.

4. Recommendations for further work: Suggests creating a database of surrogate endpoints and supporting evidence, potentially building on the Food and Drug Administration Table of Surrogate Endpoints, to assist H T A agencies and reduce validation workloads.

Navigate back to Table 2..
